# Structure-Based Identification of Allosteric Glucocerebrosidase Stabilizers from *Xylia xylocarpa* (Roxb.) Taub. for Parkinson’s Disease Using LC-MS Profiling and Computational Analysis

**DOI:** 10.3390/plants15111731

**Published:** 2026-06-03

**Authors:** Irshad Ahammed Ebrahim Thaivalappil, Aswin Mohan, Anuroopa G. Nadh, Rajesh Raju, Mohammed Gulzar Ahmed

**Affiliations:** 1 Department of Pharmaceutics, Yenepoya Pharmacy College and Research Centre, Yenepoya (Deemed to be University), Mangalore 575018, Karnataka, India; irshadahammed@yenepoya.edu.in; 2 Centre for Integrative Omics Data Science (CIODS), Yenepoya (Deemed to be University), Mangalore 575018, Karnataka, India; aswinmohan.ciods@yenepoya.edu.in (A.M.); anuroopagnadh@yenepoya.edu.in (A.G.N.); rajeshraju@yenepoya.edu.in (R.R.)

**Keywords:** Parkinson’s disease, *Xylia xylocarpa*, glucocerebrosidase, α-synuclein, LC-QTOF-MS, molecular docking, allosteric modulator

## Abstract

Parkinson’s disease is strongly linked to lysosomal dysfunction, particularly reduced activity of glucocerebrosidase (GCase) encoded by the GBA1 gene. Stabilizing GCase using small-molecule modulators represents a promising therapeutic strategy. In this study, phytochemicals from *Xylia xylocarpa* (Roxb.) Taub., a medicinal plant with reported neuroprotective potential, were profiled using LC-QTOF-MS and evaluated as GCase stabilizers through an integrated computational approach. LC-MS analysis in positive and negative modes tentatively identified 19 metabolites, of which 13 low-molecular-weight compounds (<500 Da) were selected for molecular docking against human GCase. Docking revealed six compounds with higher predicted binding affinity than the reference activator Pyrrolopyrazine. Pharmacokinetic screening based on Lipinski’s rule of five and ADMET predictions identified Senbusine A as a viable lead candidate. It exhibited favorable binding interactions, forming stabilizing contacts within a non-catalytic inter-monomer interface associated with structural modulation of GCase. PASS analysis suggested a high probability of neuroactive properties. Molecular dynamics simulations (200 ns) confirmed stable binding and reduced conformational fluctuations compared to apo and control systems. Overall, computational predictions identify Senbusine A as a potential pharmacological chaperone-like stabilizer of GCase, exhibiting a favorable pharmacological profile and warranting further experimental validation.

## 1. Introduction

Parkinson’s disease (PD) is a progressive neurodegenerative disorder and is recognized as one of the fastest-growing neurological conditions worldwide. Global epidemiological analyses from the Global Burden of Disease (GBD) study estimated that the global burden of PD has increased markedly over recent decades, with prevalent cases rising from approximately 3.15 million in 1990 to 11.77 million in 2021 (2.74-fold increase), while incident cases increased from 0.42 million to 1.34 million during the same period (2.20-fold increase) [1]. Projections further indicate that the global burden is likely to rise substantially in the coming decades, largely driven by increasing life expectancy and demographic aging.

Clinically, PD manifests through a range of motor symptoms, including bradykinesia, resting tremor, rigidity, and postural instability, accompanied by diverse non-motor manifestations such as cognitive impairment, autonomic dysfunction, and sleep disturbances. These symptoms primarily arise from the progressive degeneration of dopaminergic neurons in the substantia nigra pars compacta, resulting in decreased dopamine levels in the striatum and disruption of basal ganglia circuitry [2]. At the neuropathological level, PD is characterized by the presence of Lewy bodies and Lewy neurites, intracellular inclusions largely composed of aggregated α-synuclein [3]. The accumulation and propagation of misfolded α-synuclein and lysosomal dysfunction are widely regarded as central events in PD pathogenesis.

Among the genetic risk factors associated with PD, mutations in the *GBA* gene, which encodes the lysosomal enzyme glucocerebrosidase (GCase), represent the most common inherited contributor. Heterozygous *GBA* mutations are present in approximately 5–15% of PD patients, although prevalence varies across populations [4]. GCase catalyzes the hydrolysis of glucosylceramide (GlcCer) into glucose and ceramide, thereby maintaining cellular glycosphingolipid homeostasis. Reduced GCase activity leads to the accumulation of glycosphingolipid substrates and has been strongly implicated in the pathogenesis of both Gaucher disease and PD [5]. In PD, diminished GCase activity is closely associated with lysosomal dysfunction and progressive α-synuclein aggregation.

Accumulation of glycosphingolipids resulting from impaired GCase activity plays a critical role in regulating α-synuclein homeostasis. Elevated glucosylceramide levels stabilize soluble oligomeric intermediates of α-synuclein and promote their aggregation into neurotoxic species [6]. Similarly, increased glucosyl sphingosine concentrations facilitate the formation of fibrillar and aggregation-prone α-synuclein assemblies, linking altered lipid metabolism directly to pathological protein aggregation [7]. A bidirectional pathogenic feedback loop between GCase deficiency and α-synuclein accumulation has also been described [8]. In this model, reduced enzyme activity enhances lipid substrate accumulation and α-synuclein aggregation, while elevated α-synuclein levels further impair GCase function through direct interaction at lysosomal membranes and disruption of intracellular trafficking pathways [9]. Since diminished GCase activity in PD is primarily linked to protein misfolding and defective intracellular trafficking, targeting conformational stabilization of the enzyme has emerged as a promising therapeutic strategy for restoring lysosomal function and reducing α-synuclein pathology.

GCase is synthesized in the endoplasmic reticulum (ER) and transported to lysosomes through interaction with lysosomal integral membrane protein-2 (LIMP-2), the primary trafficking receptor responsible for lysosomal targeting of the enzyme [10]. Within lysosomes, optimal catalytic activity depends on interactions with cofactors, including phosphatidylserine-rich membrane environments and the lysosomal activator protein saposin C, which promotes extraction and solubilization of glycosphingolipid substrates for enzymatic degradation [11]. Proper folding, trafficking, and lysosomal localization of GCase are therefore essential for maintaining lipid homeostasis. Mutations such as N370S and L444P disrupt correct folding and maturation of GCase, leading to ER retention, activation of the unfolded protein response (UPR), and increased expression of stress markers, including BiP, calnexin, and CHOP [12]. Misfolded enzyme variants are frequently targeted for endoplasmic reticulum-associated degradation (ERAD) through the ubiquitin-proteasome pathway, preventing their transport to lysosomes and resulting in further accumulation of lipid substrates such as glucosylceramide, glucosylsphingosine, and sphingosine [13].

Structurally, human GCase is a 497-residue glycoside hydrolase comprising three domains, including a central catalytic TIM-barrel domain (Domain III) flanked by two auxiliary β-sheet domains (Domains I and II) that contribute to structural stability and substrate recognition. In addition to the catalytic residues Glu235 and Glu340, surrounding amino acids including Asp127, Asn234, His255, Trp179, Tyr313, and Trp381 participate in substrate binding and stabilization of the catalytic pocket [14]. Because impaired folding rather than catalytic incompetence represents a major pathogenic mechanism in *GBA1*-associated PD, therapeutic strategies increasingly focus on stabilizing conformationally competent enzyme structures.

Conventional pharmacological chaperones such as isofagomine stabilize mutant GCase by binding to the catalytic site and improving lysosomal trafficking; however, active-site engagement may lead to partial competitive inhibition at higher concentrations, thereby limiting therapeutic effectiveness [15]. Consequently, increasing attention has shifted toward allosteric stabilization strategies that target regulatory regions distal to the catalytic pocket. Allosteric modulators can enhance conformational flexibility, promote correct folding, and improve lysosomal localization of mutant GCase without interfering directly with substrate turnover [16]. Structure-based identification of small molecules capable of interacting with such regulatory pockets, therefore, can be considered as a promising approach for restoring enzyme stability and mitigating lipid-mediated α-synuclein pathology in Parkinson’s disease.

Natural products constitute a rich reservoir of structurally diverse bioactive scaffolds with significant therapeutic potential [17,18], particularly for the modulation of neurological disease targets [19,20]. *Xylia xylocarpa* (Roxb.) Taub. has attracted increasing attention as a medicinal plant due to its diverse phytochemical composition, including flavonoids, tannins, alkaloids, saponins, phenolic compounds, triterpenoids, and phytosterols such as lupeol and β-sitosterol [21,22]. These metabolites are widely associated with antioxidant, anti-inflammatory, and neuroprotective activities relevant to neurodegenerative disorders. Previous studies have reported significant acetylcholinesterase and butyrylcholinesterase inhibitory activity of *X. xylocarpa* extracts, together with improvement of scopolamine-induced memory impairment in experimental models, supporting its potential role in cognitive enhancement and neurological modulation [23]. In addition, antioxidant phenolics such as catechin derivatives, epiafzelechin oligomers, and gallic acid identified from this species further support its relevance as a source of bioactive compounds targeting pathways associated with Parkinson’s disease and related neurodegenerative conditions [21,24]. Accordingly, the present study was designed to identify potential allosteric modulators of GCase from *X. xylocarpa*, to discover candidate molecules capable of enhancing enzyme stability and activity without directly occupying the catalytic pocket.

To systematically identify potential lead candidates, the present study employed an integrated workflow combining phytochemical profiling with structure-based computational analysis. Initially, secondary metabolites present in *X. xylocarpa* were characterized using LC-QTOF-MS profiling to generate an experimentally derived library of natural compounds. This compound library was subsequently subjected to virtual screening against GCase to identify potential allosteric binders capable of stabilizing enzyme conformation. The top-ranked candidates were further evaluated through in silico pharmacokinetic and drug-likeness analyses to assess their therapeutic suitability. The selected promising compound was then subjected to molecular dynamics simulations to examine interaction stability and its conformational effects on the protein.

## 2. Materials and Methods

### 2.1. Plant Authentication and Extract Preparation

Leaves of *X. xylocarpa* were collected from the Kasaragod Forest range, Kerala, India, and taxonomically authenticated by Dr. H. S. Shenoy, Head of the Botany Division and Principal Scientist, Medicinal Garden, Pilikula, Mangalore, Karnataka, India. A voucher specimen (No. 5303) was deposited in the PND Herbarium for future reference. The collected leaves were washed, shade-dried, and pulverized into a fine powder, and approximately 100 g of the powdered material was subjected to Soxhlet extraction using 500 mL of methanol (Merck, Germany) for 6–8 h (10–12 cycles) until the solvent in the siphon tube became colorless, with the extraction temperature maintained at the boiling point of methanol (~65 °C). The extract was concentrated under reduced pressure using a rotary evaporator until complete removal of methanol, yielding a dry crude methanolic extract, which was stored in an airtight container at room temperature until further analysis. Methanol was selected as the extraction solvent due to its ability to efficiently solubilize structurally diverse polar and semi-polar phytoconstituents, thereby enabling broad metabolite coverage and enhanced detection sensitivity in LC-QTOF-MS-based phytochemical profiling [25]. The percentage yield of the extract was calculated using the formula:

Yield (%) = (Weight of crude extract × 100)/Weight of dried plant material.

#### LC-QTOF-MS Analysis

LC-QTOF-MS profiling of the plant extract was performed in both positive and negative electrospray ionization modes, and the detected metabolites were tentatively annotated based on accurate mass matching with available spectral libraries. For molecular docking against human GCase, a rational compound selection strategy was adopted to ensure biological relevance, chemical reliability, and structural suitability for interaction with the enzyme binding site implicated in Parkinson’s disease-associated GBA1 dysfunction. Initially, signals corresponding to non-plant-derived analytical contaminants, including plasticizers, phthalates, maleates, surfactants, pharmaceutical residues, and compounds originating from extractables/leachables and toxicology or screening-library matches, were excluded. Fatty acids and long-chain aliphatic compounds, which typically exhibit nonspecific hydrophobic interactions and limited pharmacological chaperone potential, were also omitted. From the remaining dataset, only bona fide phytochemicals were considered and prioritized based on the following criteria:

(i) tentative LC-MS annotation with acceptable mass accuracy and reproducible retention behavior
(ii) relatively high signal intensity/abundance in the chromatogram
(iii) appropriate molecular size compatible with access to the GCase binding pocket or adjacent stabilizing regions

Very large and highly glycosylated molecules (e.g., bulky saponins and complex glycosides) and small, simple terpenes lacking sufficient functionality for specific binding were further excluded to minimize nonspecific docking artifacts. A molecular-weight filter (<500 Da) was additionally applied to prioritize small drug-like metabolites suitable for pharmacological chaperone interaction with GCase.

### 2.2. Target Preparation

The X-ray crystal structure of wild-type human GCase, resolved at 1.85 Å and co-crystallized with a pyrrolo [2,3-b] pyrazine-based activator [26], was obtained from the Protein Data Bank (PDB) with PDB ID 6T13. Protein preparation was performed using the Protein Preparation Wizard in Schrödinger Maestro (Version 14.1.138) (Release 2024-3) [27]. During pre-processing, bond orders were assigned, and hydrogen atoms were added, while crystallographic artifacts, redundant heteroatoms, and non-essential chains were removed. Missing side chains and loop regions were reconstructed using the Prime module to generate a structurally complete receptor suitable for downstream computational analysis. Protonation states of ionizable residues were assigned at physiological pH (7.0 ± 0.2) using Epik (Version 5.2) followed by optimization of the hydrogen-bonding network. The prepared structure was subsequently subjected to restrained energy minimization with the OPLS4 force field until convergence was achieved, using a heavy-atom root-mean-square deviation (RMSD) threshold of 0.3 Å.

### 2.3. Molecular Docking

The co-crystallized activator Pyrrolopyrazine, a reported GCase activator, was used as a reference control ligand to validate the docking protocol and to provide a comparative baseline for evaluating the interaction potential of the selected phytochemicals with the enzyme. Structural inspection of the complex revealed a ligand-binding cavity located at an inter-monomer interface, formed by residues contributed from two adjacent chains. The docking protocol was validated through re-docking of the native co-crystallized ligand into the prepared receptor using identical docking parameters. The accuracy of the protocol was assessed by calculating the root mean square deviation (RMSD) between the predicted docking pose and the experimentally determined ligand conformation. An RMSD value of ≤2.0 Å was considered indicative of a reliable docking procedure capable of reproducing the experimentally observed binding orientation. Re-docking of the co-crystallized ligand Pyrrolopyrazine confirmed that the RMSD between the predicted and experimental binding poses was below 2.0 Å, validating the reliability of the docking protocol used for subsequent screening.

Phytochemical structures were prepared using the LigPrep module of Schrödinger to generate energetically optimized three-dimensional conformations. During ligand preparation, possible ionization states were assigned, and stereoisomers were retained where applicable. Molecular docking was performed using the Glide Extra Precision (XP) protocol. The docking algorithm evaluated multiple ligand poses within the defined grid, and the best binding orientation was selected based on intermolecular interactions with key residues and on GlideScore, which approximates binding free energy by considering hydrophobic, electrostatic, hydrogen-bonding, and van der Waals interactions.

### 2.4. ADMET and Lipinski Filtering

Physicochemical and pharmacokinetic properties of the top-ranked compounds were evaluated using the QikProp module of Schrödinger (Version M16.1), which predicts key absorption, distribution, and drug-likeness descriptors, including molecular weight, lipophilicity, aqueous solubility, blood-brain barrier (BBB) penetration, and human oral absorption. Physicochemical descriptors predicted using the QikProp module included molecular weight (MW), octanol-water partition coefficient (logP), number of hydrogen bond donors (HBD), number of hydrogen bond acceptors (HBA), predicted aqueous solubility (QPlogS), predicted BBB permeability (QPlogBB), and predicted human oral absorption (HOA). Lipinski’s rule of five filtering was applied using the criteria MW ≤ 500 Da, logP ≤ 5, HBD ≤ 5, and HBA ≤ 10 to prioritize structurally suitable drug-like candidates for further evaluation. Hepatotoxicity risk and additional pharmacokinetic parameters were predicted using ADMETlab 3.0, which applies machine-learning-based models trained on experimentally validated datasets. Output values were interpreted according to the platform’s recommended classification ranges, with hepatotoxicity probability values <0.30 considered low risk.

### 2.5. Neurological Activity Prediction Using PASS

The possible neurological activity profile of promising candidates was predicted using PASS (Prediction of Activity Spectra for Substances) online software, which estimates the probability of various pharmacological activities based on structure-activity relationship analysis (https://way2drug.com/PassOnline/) (accessed on 10 February 2026). The predicted activities were interpreted based on probability values (Pa > Pi), indicating a higher likelihood of the compound exhibiting the corresponding biological effect [28].

### 2.6. Molecular Dynamics Simulations

Molecular dynamics (MD) simulations were performed using the GROMACS package [29] (version 2024.1) employing the CHARMM36 force field to assess the structural stability of the protein-ligand, protein-drug, and apo protein systems. Topology parameters for the ligand and reference compound were generated through the CGenFF server, while the protein topology was prepared using the pdb2gmx module. Each system was placed in a triclinic simulation box solvated with the TIP3P water model, and electrical neutrality was achieved by adding appropriate Na^+^ and Cl^−^ counterions. Energy minimization was conducted using the steepest descent algorithm until the maximum force fell below 1000 kJ mol^−1^ nm^−1^. System equilibration proceeded in two phases: an NVT ensemble at 300 K maintained with the V-rescale thermostat for 200 ps, followed by an NPT ensemble at 1 bar controlled using the Parrinello-Rahman barostat for 500 ps. Subsequently, a 200 ns production simulation was performed under periodic boundary conditions. Bond lengths were constrained using the LINCS algorithm with a 2 fs integration time step, and long-range electrostatic interactions were treated using the Particle Mesh Ewald method. Trajectory stability and conformational behavior were evaluated through analyses of RMSD, RMSF, radius of gyration (Rg), solvent-accessible surface area (SASA), and hydrogen bonding patterns.

## 3. Results

### 3.1. LC-QTOF-MS Analysis

**Figure 1:**
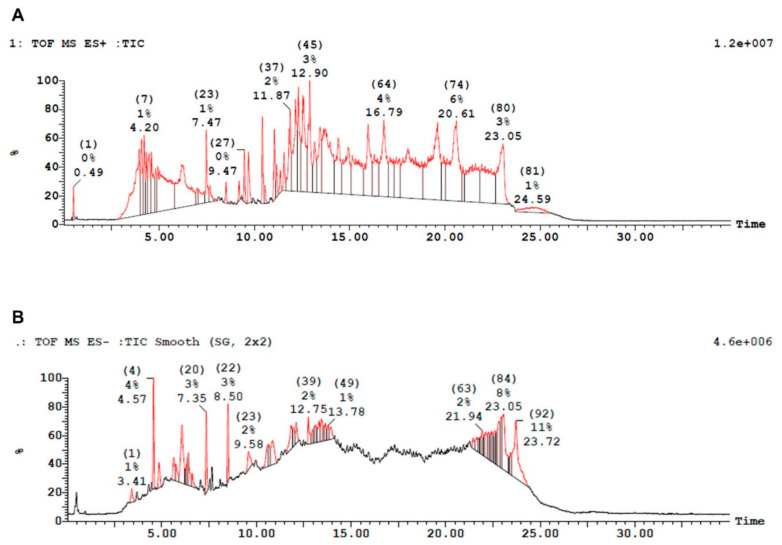
LC-QTOF-MS total ion chromatograms of *X. xylocarpa* extract recorded in (**A**) positive ionization mode (ESI+), (**B**) negative ionization mode (ESI−).

The extraction yield of the methanolic extract of *X. xylocarpa* (MEXX) was 7.37% (*w*/*w*) relative to the initial dry plant material. LC-QTOF-MS analysis of MEXX in both positive and negative ionization modes revealed multiple well-resolved chromatographic peaks throughout the run, indicating the presence of structurally diverse phytoconstituents within the extract (Figure 1). Following multi-step filtering based on signal abundance, removal of duplicate features, and exclusion of signals corresponding to analytical contaminants (extractables/leachables, plasticizers, and toxicology or screening-library matches), a total of 19 metabolites were tentatively annotated from the combined positive and negative ionization mode datasets before downstream filtering for docking suitability (Supplementary Table S1). It should be noted that metabolite identification was based on accurate mass matching with spectral libraries and therefore represents tentative annotation without confirmatory MS/MS fragmentation analysis or comparison with authentic reference standards.

Among these, most low-molecular-weight secondary metabolites, including flavonoids, alkaloids, terpenoids, sterols, and phenolic ketones, were preferentially detected in the positive ionization mode, whereas fatty acids and other polar constituents were predominantly observed in the negative ionization mode. Following the application of a molecular-weight filter (<500 Da) to prioritize structurally diverse small drug-like molecules, 13 representative phytochemicals were selected for further analysis (Table 1). These compounds comprised flavonoids/phenolics (Licoricidin, Methyl nigakinone, Vitetrifolin C, Tokinolide B, Acetoxy-[10]-gingerol), an alkaloid (Senbusine A), terpenoid/sterol derivatives (Bufalin, Cerevisterol, 12-Oxoarundoin, ent-12α,16-epoxy-pimarene derivative), and fatty acid derivatives (14,17-octadecadienoic acid and 2-cyclopentene-1-undecanoic acid). This final set of compounds was subsequently used for structure-based virtual screening against human GCase to evaluate their potential as pharmacological chaperones relevant to Parkinson’s disease.

### 3.2. Molecular Docking

**Figure 2:**
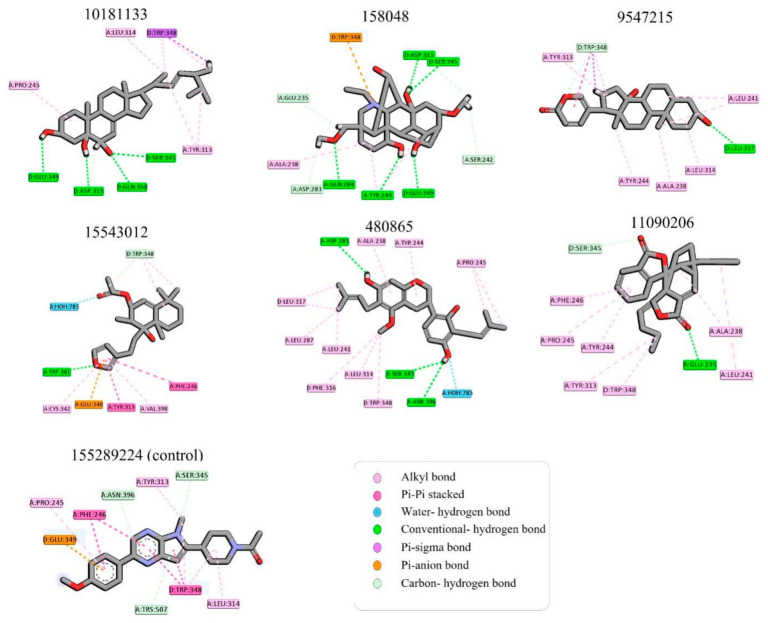
Interaction analysis of selected phytochemicals from *X. xylocarpa* with the glucocerebrosidase binding cleft compared with the reference activator Pyrrolopyrazine.

Docking analysis indicated that 6 of the 13 selected compounds showed higher predicted binding affinity toward GCase than the reference activator, Pyrrolopyrazine. The Glide docking scores of these compounds are summarized in Table 2. Although docking scores provide a useful estimate of relative binding affinity within the defined interface pocket, compound prioritization in the present study was not based solely on Glide score ranking. Docking score differences alone do not necessarily reflect overall therapeutic suitability. Therefore, subsequent prioritization incorporated predicted interaction profile, pharmacokinetic behavior, and Lipinski-based drug-likeness assessment to identify compounds with a more balanced profile for central nervous system-relevant applications. Most compounds exhibited favorable binding interactions within the GCase binding cleft and adopted binding orientations similar to that of the control. Figure 2 shows the binding mode of these compounds and the control activator against GCase. Key interacting residues and the nature of interactions are listed in Table 3.

Two-dimensional interaction analysis showed that the selected phytochemicals formed multiple stabilizing hydrogen-bonding and hydrophobic interactions with residues located in the non-catalytic binding region of GCase (Domain III), including Tyr313, Trp348, Ser345, and Glu349. Several compounds exhibited interaction patterns comparable to those of the reference activator Pyrrolopyrazine, indicating favorable binding within the regulatory pocket associated with enzyme stabilization.

### 3.3. ADMET and Lipinski Filtering

The 6 selected compounds were further evaluated for their drug-likeness and pharmacokinetic properties using Lipinski’s rule of five and in silico ADMET prediction analyses (Table 4).

**Figure 3:**
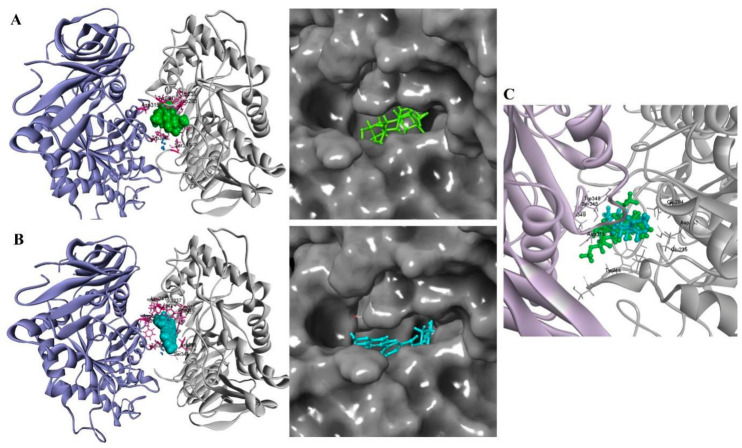
Comparative molecular docking analysis of Senbusine A and Pyrrolopyrazine (control) at the inter-monomer interface of glucocerebrosidase. (**A**) Binding pose of Senbusine A (green) within the interface cavity formed by adjacent GCase chains A and D. (**B**) Binding pose of the control compound Pyrrolopyrazine (cyan) at the same binding site. The middle panel shows an enlarged surface representation of the binding pocket. (**C**) Detailed view of ligand interactions with key interface residues showing stabilization of the binding pocket.

Among the evaluated compounds, Senbusine A (PubChem ID: 158048) demonstrated the most favorable pharmacokinetic profile, showing no violations of Lipinski’s rule of five, high predicted oral absorption, good solubility, moderate serum albumin binding, low hepatotoxicity risk, and moderate BBB permeability. Based on these properties, Senbusine A was selected for subsequent analysis, including biological activity prediction and molecular dynamics simulation studies. Although Cerevisterol exhibited the highest docking score among the screened compounds, its predicted pharmacokinetic profile was less favorable than that of Senbusine A, and therefore, compound prioritization was guided by integrated docking and ADMET suitability criteria rather than binding affinity alone. Figure 3 illustrates the binding mode of Senbusine A within the GCase binding pocket in comparison with the reference activator Pyrrolopyrazine.

**Figure 4:**
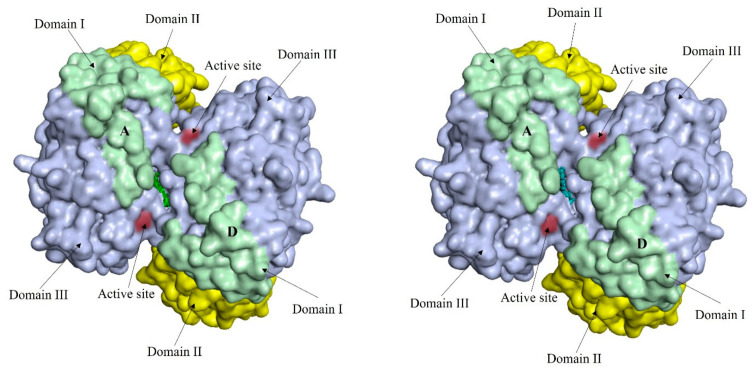
Surface representation of human glucocerebrosidase (GCase) illustrating the binding localization of Senbusine A (green) compared with the reference activator Pyrrolopyrazine (cyan) within chains A and D. Domain organization highlights that both ligands occupy a similar non-catalytic stabilizing region (Domain III) of the enzyme associated with structural modulation rather than the catalytic pocket.

The comparative binding localization of Senbusine A and the reference activator, Pyrrolopyrazine, within a non-catalytic stabilizing region of GCase across chains A and D is shown in Figure 4.

Surface mapping of ligand-binding localization revealed that Senbusine A occupies a region comparable to that of the reference activator Pyrrolopyrazine, within Domains-III, across the interface of chains A and D of GCase, positioned outside the catalytic pocket. Notably, both the control and the phytochemical were localized within a non-catalytic stabilizing region of the enzyme, suggesting a possibility of pharmacological chaperone-like mode of interaction rather than direct catalytic-site engagement. The spatial separation from the catalytic residues and higher binding affinity and similar binding mode to the control activator further support a potential role for Senbusine A in structural stabilization of GCase.

### 3.4. Neurological Activity Prediction

PASS prediction analysis indicated that Senbusine A has a probability of neuroactive pharmacological properties, including anesthetic, analgesic, and cholinergic antagonist activities, suggesting its potential as a neuroprotective agent (Table 5).

### 3.5. Molecular Dynamics Simulation

**Figure 5:**
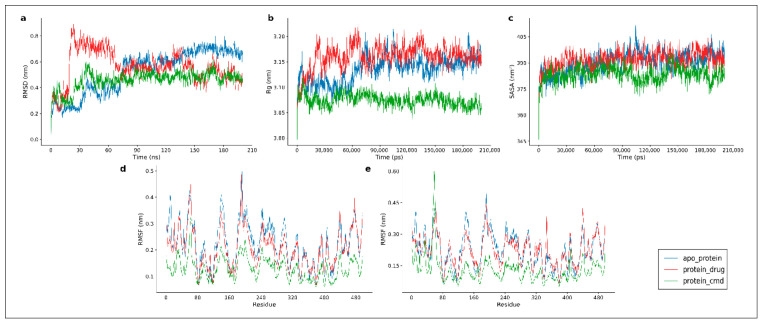
Molecular dynamics simulation profiles of glucocerebrosidase in the apo state (blue), protein-Pyrrolopyrazine (control) complex (red), and protein-Senbusine A (ligand) complex (green) over 200 ns. (**a**) Root mean square deviation (RMSD) of backbone atoms showing stabilization of the ligand-bound systems relative to the apo structure. (**b**) The radius of gyration (Rg) indicates maintenance of structural compactness during simulation. (**c**) Solvent-accessible surface area (SASA) demonstrates comparable solvent exposure across all systems. (**d**) Root mean square fluctuation (RMSF) of chain A residues illustrating reduced flexibility upon ligand binding. (**e**) Root mean square fluctuation (RMSF) of chain D residues confirms decreased residue-level fluctuations in the Senbusine A-bound complex compared with the apo and control systems.

Molecular dynamics (MD) simulations were performed to evaluate the conformational stability of the protein-ligand complexes. Three systems were simulated for 200 ns, including the apo protein, the protein-control complex (6T13-pyrrolopyrazine), and the protein-ligand complex (6T13-Senbusine A). The trajectories generated during the production phase were analyzed for root mean square deviation (RMSD), root mean square fluctuation (RMSF), radius of gyration (Rg), and solvent-accessible surface area (SASA) to assess structural stability and dynamic behavior during the simulation (Figure 5).

RMSD analysis over the 200 ns simulation exhibited the backbone behavior of the free protein, protein-ligand complex, and protein-Pyrrolopyrazine complex. The apo protein exhibited an upward trend till 80 ns and plateaued afterwards with RMSD values ranging from 0.0005005 to 0.79 nm, with a mean RMSD of 0.52 ± 0.16 nm and a final value of 0.68 nm at 200 ns. The protein-ligand complex reached the equilibrium at 40 ns, deviating between 0.4 nm and 0.6 nm till the end of the simulation, with overall RMSD values ranging between 0.0004964 and 0.61 nm, with an average RMSD of 0.45 ± 0.06 nm and a terminal value of 0.45 nm. In contrast, the protein-control complex displayed the highest RMSD fluctuations from 0.0005041 to 0.89 nm, yielding a mean RMSD of 0.56 ± 0.12 nm and a final RMSD of 0.46 nm at the end of the simulation. An initial rise to 0.89 nm followed by a downward trend in which the RMSD plot converged with the compound’s RMSD around 150 ns. The three RMSD plots exhibited convergence between 70 ns and 120 ns.

The Rg analysis indicated stable global structural compactness across all systems throughout the 200 ns simulation. The reference free protein system showed Rg values ranging from 2.998 to 3.214 nm, with a mean value of 3.135 ± 0.026 nm, increasing from an initial 2.998 nm to a final 3.147 nm. After the initial rise, the Rg plot fluctuated between 3.07 nm and 3.21 nm. Similarly, the protein-Pyrrolopyrazine complex displayed Rg values ranging from 2.999 to 3.224 nm, with a mean of 3.160 ± 0.024 nm and a final value of 3.172 nm. The 6T13-ligand complex showed a narrower distribution between 2.997 and 3.129 nm, with a mean Rg of 3.075 ± 0.012 nm and a final value of 3.057 nm. Across all trajectories, fluctuations remained within a limited range, indicating preservation of overall structural compactness during the simulation.

SASA analysis indicated stable surface exposure across all systems during the simulation. The free protein exhibited SASA values ranging from 278.54 to 301.82 nm^2^, with a mean of 289.67 ± 4.12 nm^2^ and a final value of 291.03 nm^2^. The protein-control complex displayed SASA values between 281.32 and 305.76 nm^2^, with a mean of 293.58 ± 4.95 nm^2^ and a final value of 296.14 nm^2^. In contrast, the 6T13-ligand complex showed SASA values ranging from 270.11 to 289.45 nm^2^, with a mean of 279.84 ± 3.76 nm^2^ and a final value of 278.62 nm^2^. Across all trajectories, SASA fluctuations remained within a comparable range, indicating preservation of overall solvent exposure throughout the simulation.

RMSF analysis of chain A and chain D was carried out separately. The free protein exhibited RMSF values ranging from 0.041 to 0.842 nm, with a mean fluctuation of 0.214 ± 0.118 nm. The protein-control complex showed RMSF values between 0.038 and 0.796 nm, with a mean of 0.198 ± 0.109 nm. In contrast, the 6T13-ligand complex displayed RMSF values ranging from 0.036 to 0.721 nm, with a mean value of 0.183 ± 0.097 nm. The chain D of the free protein exhibited RMSF values ranging from 0.039 to 0.865 nm, with a mean fluctuation of 0.221 ± 0.124 nm. The protein-control complex showed RMSF values between 0.037 and 0.812 nm, with a mean value of 0.206 ± 0.113 nm. The 6T13-ligand complex displayed RMSF values ranging from 0.035 to 0.748 nm, with a mean fluctuation of 0.189 ± 0.101 nm.

## 4. Discussion

The central objective of this study was to determine whether phytochemicals from *X. xylocarpa* could engage a non-catalytic regulatory interface of GCase with sufficient affinity and selectivity to function as allosteric stabilizers, an approach motivated by the well-established link between GCase misfolding, lysosomal dysfunction, and progressive α-synuclein pathology in GBA1-associated PD. Unlike catalytic-site pharmacological chaperones, which risk partial competitive inhibition at therapeutically relevant concentrations, interface-targeting ligands offer a mechanistically distinct route to restoring enzyme stability without compromising substrate turnover. Against this rationale, LC-MS-guided screening of *X. xylocarpa* metabolites followed by structure-based docking, pharmacokinetic filtering, and molecular dynamics simulation converged on Senbusine A as the lead candidate, distinguished not only by its binding affinity but by the breadth and character of its interactions within the regulatory pocket.

Initially, the chemical composition of the plant extract was characterized by LC-QTOF-MS analysis in both positive (ESI^+^) and negative (ESI^−^) electrospray ionization modes. The total ion chromatograms (TICs) revealed a complex metabolite profile with multiple well-resolved peaks across the retention time range, indicating the presence of structurally diverse secondary metabolites.

In the ESI^+^ mode, a total of ~90 chromatographic peaks were detected. In the ESI^−^ mode, approximately 95 peaks were observed. After removing signals corresponding to analytical contaminants (extractables/leachables, plasticizers, and toxicology/screening library hits) and duplicate adduct forms, a total of 19 unique plant-derived metabolites were obtained from both ionization modes combined. These included molecules belonging mainly to flavonoids, phenolic derivatives, alkaloids, terpenoids, sterols, and glycosides, covering a molecular weight range of approximately 120–920 Da. Among these, most low-molecular-weight secondary metabolites were preferentially detected in the positive ion mode, whereas the negative ion mode was dominated by fatty acids and highly polar glycosides. For subsequent in silico studies, an additional molecular weight filter of <500 Da was applied to focus on small, drug-like molecules, which resulted in a list of 13, low-molecular-weight natural compounds that were considered for molecular docking against human GCase.

The co-crystallized activator, Pyrrolopyrazine, was employed as the reference ligand for subsequent computational analyses. Structural inspection of the complex revealed a ligand-binding cavity located at an inter-monomer interface, formed by residues contributed from two adjacent chains. To preserve the structural integrity of this composite binding site, chains A and D, which constitute the crystallographic interface forming the pocket, were retained for docking studies. Docking analysis indicated that most of the screened compounds occupy the inter-monomer interface cavity observed in the crystal structure of GCase, engaging residues contributed by both adjacent chains. In the context of PD, where reduced GCase activity arises from misfolding and structural destabilization, interface-binding ligands may reinforce inter-domain packing and reduce conformational fluctuations. The predominance of hydrophobic and aromatic stacking interactions observed across multiple compounds supports the hypothesis that these molecules could function as pharmacological stabilizers, promoting structural integrity and potentially enhancing lysosomal trafficking of GCase.

Docking analysis revealed that 6 compounds, Cerevisterol, Senbusine A, Vitetrifolin C, Licoricidin, Bufalin, and Tokinolide B demonstrated higher affinity towards the GCase than the control drug (−8.01 Kcal/mol). Among these, Cerevisterol showed the highest affinity (−10.29 Kcal/mol). Although GCase functions predominantly as a monomer under lysosomal conditions, the identified interface pocket represents a structurally contiguous region that contributes to local conformational stability of the catalytic TIM-barrel domain. Ligand binding at the inter-monomer interface may stabilize adjacent secondary structural elements surrounding the catalytic TIM-barrel domain and preserve active-site geometry without directly blocking catalysis. Ligand binding across this composite surface may restrict unfavorable domain fluctuations, stabilize secondary structural elements adjacent to the catalytic residues (Glu235 and Glu340), and preserve the geometry of the active-site architecture. It was observed that the docked compounds demonstrated stable accommodation within the inter-monomer interface cavity of GCase, forming multiple interactions with residues contributed by both chains back Recurrent engagement of residues such as Tyr313, Trp348, Phe246, Pro245, Ala238, Glu235, Asp315, Ser345, and Asn396 indicates that the ligands occupy a structurally conserved pocket bridging the catalytic TIM-barrel domain and adjacent structural elements. Notably, several compounds exhibited π-π stacking and π-sigma interactions with aromatic residues (Tyr313, Trp348, Phe246), suggesting stabilization of hydrophobic packing within the interface region. Concurrent hydrogen bonding and conventional hydrogen bond formation with residues such as Glu235, Asp315, Ser345, and Asn396 may contribute to conformational rigidity and local electrostatic stabilization. Importantly, Glu235, one of the catalytic residues, appears in interaction profiles of Senbusine A and Tokinolide B, but without steric obstruction of the catalytic dyad, supporting a non-inhibitory binding mode. This interaction pattern suggests potential allosteric stabilization rather than competitive inhibition.

Although Cerevisterol exhibited a slightly higher Glide score than Senbusine A, docking score differences alone do not necessarily determine lead suitability, as they primarily reflect relative binding affinity within a defined structural framework. Therefore, compound prioritization in the present study incorporated an integrated evaluation of interaction characteristics together with predicted pharmacokinetic behavior and Lipinski-based drug-likeness properties. Within this framework, Senbusine A satisfied all evaluated criteria and demonstrated favorable predicted oral absorption, acceptable blood-brain barrier permeability, and low hepatotoxicity risk, supporting its selection as the most suitable candidate for further molecular dynamics analysis and biological activity prediction relevant to glucocerebrosidase stabilization. Molecular docking results further indicated that Senbusine A formed stronger and more extensive interactions within the inter-monomer interface cavity of GCase compared to other compounds, and the control compound, Pyrrolopyrazine, suggesting its potential role as an allosteric stabilizer of GCase. It is evident from the interaction that Senbusine A exhibited a stable binding orientation and formed multiple hydrogen bonds with key residues such as Asp315 and Ser345, along with additional interactions with Glu235, Gln284, Asp283, and Glu349, contributing to the strong stabilization of the binding pocket. Hydrophobic and aromatic interactions with Trp348 and Tyr244 further enhanced ligand binding. Importantly, interaction with the catalytic residue Glu235 occurred without blocking the catalytic site, indicating a non-competitive binding mode consistent with allosteric regulation. In contrast, the control compound Pyrrolopyrazine showed fewer interactions and mainly formed hydrophobic and π-π interactions with aromatic residues such as Tyr313, Phe246, and Trp348, with limited hydrogen bonding. This suggests weaker binding stability compared to Senbusine A.The comparative binding mode and localization of Senbusine A and Pyrrolopyrazine (Figure 3 and Figure 4) show that both compounds occupy the same binding site and adopt a similar orientation. However, docking parameters suggest that Senbusine A exhibited stronger interactions within the pocket, supporting its potential role as an allosteric activator compared to the control. Although the present structure-based workflow identified Senbusine A as a promising candidate capable of stabilizing GCase at a non-catalytic interface region, experimental validation through enzymatic activity assays and lysosomal trafficking studies will be necessary to confirm its functional effects on GCase. In particular, in vitro GCase activity assays using fluorogenic substrates and cellular models of GBA1-associated lysosomal dysfunction could help determine whether the predicted interaction profile translates into restoration of enzyme stability and modulation of α-synuclein-associated pathological processes under physiological conditions.

PASS prediction result showed that Senbusine A possesses a high probability of neuroprotective activities, including anesthetic (Pa = 0.97), local anesthetic (Pa = 0.92), and analgesic (Pa = 0.89) activities, along with predicted acetylcholine receptor antagonist and cholinergic antagonist effects (Pa ≈ 0.84–0.86). Notably, these predicted activities, together with its moderate BBB permeability observed in ADMET analysis, suggest that Senbusine A may exert CNS-relevant effects. Such properties support its potential role as a neuroactive stabilizing ligand capable of modulating GCase function in the context of PD-associated GBA1 dysfunction. The observation that Senbusine A from *X. xylocarpa* exhibited cholinergic antagonist effects further supports previous experimental evidence showing that extracts of this plant display cholinesterase inhibitory activity and cognitive-enhancing effects [23].

Further molecular simulation analysis was carried out to assess the binding stability of Senbusine A with GCase based on the thermodynamic parameters.

RMSD analysis over the 200 ns trajectory revealed distinct conformational behavior across the three systems. The apo protein underwent a progressive rise in backbone deviation until approximately 80 ns before plateauing, reflecting the inherent flexibility of the unliganded enzyme. The protein-Pyrrolopyrazine complex reached equilibrium earlier, around 40 ns, and maintained moderate deviation thereafter. Notably, the protein-Senbusine A complex displayed the lowest overall RMSD throughout the trajectory, with convergence of all three systems observed between 80 and 120 ns. The continued divergence of the apo protein from both ligand-bound systems beyond this point highlights the role of small-molecule binding in suppressing large-scale backbone fluctuations—a behavior particularly relevant in the context of GCase misfolding, where conformational instability drives ER retention and lysosomal trafficking failure.

Rg analysis reinforced these findings. The apo protein and protein-Pyrrolopyrazine complex followed broadly parallel compactness trajectories from approximately 60 ns onward, both exhibiting larger and more variable Rg values than the Senbusine A-bound system. The protein-Senbusine A complex maintained a consistently lower and less variable Rg (mean 3.075 ± 0.012 nm), indicating that ligand binding at the inter-monomer interface imposes a measurable constraint on global domain packing. This degree of compactness, exceeding that achieved by the reference activator, suggests that Senbusine A engages the regulatory pocket with greater structural consequence.

SASA values across all three systems remained within a broadly comparable range, but the protein—Senbusine A complex consistently exhibited the lowest solvent-exposed surface area (mean 279.84 ± 3.76 nm^2^), consistent with tighter domain closure. This reduction in solvent accessibility, corroborated by the Rg data, supports a model in which Senbusine A binding promotes a more compact and protected enzyme conformation.

RMSF analysis of both chains A and D revealed a stepwise reduction in residue-level flexibility in the order: apo > protein-Pyrrolopyrazine > protein-Senbusine A. The magnitude of fluctuation peaks was consistently attenuated in the Senbusine A-bound complex across both chains, with chain D showing slightly higher baseline flexibility than chain A in all systems—a pattern likely reflecting the peripheral role of chain D in forming the composite interface pocket. The preferential dampening of fluctuations in the Senbusine A-bound system, relative even to the established activator, supports the hypothesis that this compound stabilizes not only the immediate binding interface but also the broader domain architecture surrounding the catalytic TIM-barrel.

Overall, the present study highlights the potential of phytochemicals from *X. xylocarpa* as modulators of GCase stability through interaction with a non-catalytic regulatory region associated with conformational stabilization of the enzyme. Among the screened compounds, Senbusine A demonstrated a favorable binding affinity together with the most balanced interaction profile, pharmacokinetic properties, and predicted neuroactive potential, supported by stable conformational behavior during molecular dynamics simulations. Localization of the ligand within an inter-domain interface region rather than the catalytic pocket further supports a pharmacological chaperone-like mechanism that may enhance enzyme stability without interfering with substrate turnover. Given the central role of GCase dysfunction in GBA1-associated PD, these findings suggest that Senbusine A can be considered a promising candidate for further experimental validation as a potential allosteric stabilizer of GCase. Future investigations should include confirmatory structural characterization of the detected metabolites using tandem MS fragmentation and authentic reference standards to strengthen compound-level annotation confidence. In addition, a detailed toxicological evaluation of Senbusine A will be necessary, particularly because alkaloids may exhibit dose-dependent pharmacological and off-target effects that influence central nervous system safety. Comprehensive in vitro cytotoxicity assays, lysosomal trafficking studies, and enzyme activity measurements should therefore be performed to determine whether the predicted stabilizing interaction with glucocerebrosidase translates into functional restoration of enzyme activity. Furthermore, in vivo studies in Parkinson’s disease-relevant experimental models will be required to assess pharmacokinetic behavior, blood-brain barrier penetration, and potential neuroprotective efficacy under physiological conditions.

### Study Limitations

Despite the promising findings obtained in this study, several limitations should be acknowledged. First, the phytochemical identification was based on tentative LC-MS annotation using accurate mass matching without confirmatory MS/MS fragmentation or reference standard validation, which may affect structural certainty of the reported metabolites. Second, the interaction profiles and stabilizing effects of the selected compounds were derived from computational predictive analysis and therefore require experimental validation through enzymatic and cellular assays to confirm their influence on GCase folding, stability, and enzymatic activity. In particular, the absence of in vitro GCase activity measurements and disease-relevant cellular validation for Senbusine A represents an important limitation of the present study. Future investigations employing biochemical enzyme assays and lysosomal trafficking models will be necessary to confirm its pharmacological chaperone-like stabilizing effects on GCase. In addition, the predicted pharmacokinetic and neuroactive properties obtained from ADMET and PASS analyses represent in silico estimates that may not fully reflect biological behavior under physiological conditions. Finally, the present study focused primarily on structural stabilization at a regulatory interface region of GCase and did not evaluate downstream functional outcomes such as lysosomal trafficking efficiency or modulation of α-synuclein aggregation. Accordingly, further biochemical, cellular, and in vivo investigations are necessary to establish the therapeutic potential of Senbusine A as an allosteric stabilizer of GCase in PD.

## 5. Conclusions

Based on computational predictive assessment, this study demonstrates that phytochemicals from *X. xylocarpa* can interact with a regulatory interface region of glucocerebrosidase associated with conformational stabilization of the enzyme, suggesting an allosteric pharmacological chaperone-like mechanism in the context of GBA1-associated Parkinson’s disease. Among the screened metabolites, Senbusine A showed the most favorable binding orientation, pharmacokinetic profile, predicted neuroactive properties, and dynamic stability within the inter-domain interface pocket without interfering with the catalytic dyad. Molecular dynamics simulations further indicated reduced structural fluctuations of the protein-ligand complex relative to both apo and reference activator systems, consistent with enhanced conformational stability of GCase.

Taken together, these findings highlight Senbusine A as a promising candidate for modulating GCase structural integrity and identify *X. xylocarpa* as a potential source of allosteric-stabilizing phytochemicals targeting lysosomal dysfunction in Parkinson’s disease. Experimental validation in biochemical and cellular models will be necessary to confirm its effects on enzyme folding, lysosomal trafficking, and α-synuclein-associated pathology. Future validation through in vitro enzymatic assays and Parkinson’s disease-relevant cellular models will be essential to confirm the predicted stabilizing activity of Senbusine A on GCase function.

## Figures and Tables

**Table 1:** LC-QTOF-MS-annotated phytochemicals from *X. xylocarpa* selected for molecular docking against human glucocerebrosidase.

Sl. No.	Compound Name	RT (min)	Observed *m*/*z*	Adduct	Ion Mode	Molecular Weight (Da)	Compound Class
1.	Licoricidin	4.93	447.21	+Na	ESI+	424.22	Isoflavonoid
2.	Vitetrifolin C	6.18	399.19	+K	ESI+	360.23	Diterpenoid
3.	Tokinolide B	4.81	403.19	+Na	ESI+	380.20	Sesquiterpene lactone
4.	Acetoxy-[10]-gingerol	12.90	413.26	+Na	ESI+	390.27	Phenolic ketone
5.	Senbusine A	13.73	441.29	+NH_4_	ESI+	423.26	Alkaloid
6.	Bufalin	10.43	425.21	+K	ESI+	386.25	Steroidal terpenoid
7.	Cerevisterol	16.07	430.34	+H	ESI+	430.34	Sterol
8.	Methyl nigakinone	4.59	319.05	+K	ESI+	280.09	Naphthoquinone
9.	Hexadecyl ferulate	13.73	419.31	+H	ESI+	418.31	Phenolic ester
10.	12-Oxoarundoin	14.42	454.38	+K/+Na	ESI+	454.38	Triterpenoid derivative
11.	ent-12α,16-Epoxy-pimarene derivative	11.37	337.24	+H	ESI+	336.23	Diterpenoid
12.	14,17-Octadecadienoic acid	13.97	325.24	+HCOO	ESI−	280.24	Fatty acid
13.	2-Cyclopentene-1-undecanoic acid	10.66	311.22	+CH_3_COO	ESI−	252.21	Fatty acid

**Table 2:** Selected phytochemicals from *Xylia xylocarpa* (Roxb.) Taub.exhibiting higher predicted binding affinity toward glucocerebrosidase than the reference activator Pyrrolopyrazine.

Sl. No	Compound Name	PubChem ID	Glide Score (Kcal/mol)
1.	Cerevisterol	10181133	−10.29
2.	Senbusine A	158048	−9.98
3.	Vitetrifolin C	15543012	−9.72
4.	Licoricidin	480865	−9.27
5.	Bufalin	9547215	−9.08
6.	Tokinolide B	11090206	−8.66
7.	Pyrrolopyrazine (control)	155289224	−8.01

**Table 3:** Residue-level interaction profile of selected *Xylia xylocarpa* (Roxb.) Taub.phytochemicals and the reference ligand Pyrrolopyrazine within the glucocerebrosidase regulatory binding pocket based on Glide XP docking analysis.

PubChem ID	Compounds	Conventional Hydrogen-Bond Interactions	π-π/Aromatic Interactions	Hydrophobic Contacts	Electrostatic Interactions	Carbon-Hydrogen Bond	Water-Hydrogen Bond
10181133	Cerevisterol	SER 345, GLN 350, ASP 315, GLU 349	TRP 348	PRO 245, LEU 314, TYR 313			
158048	Senbusine A	ASP 315, SER 345, GLU 349, TYR 244, GLN 284		ALA 238	TRP 348,	SER 242, ASP 283, GLU 235	
9547215	Bufalin	LEU 317		TYR 313, LEU 241, LEU 314, ALA 238, TYR 244		TRP 348	
15543012	Vitetrifolin C	TRP 381	TYR 313, PHE 246	VAL 398, CYS 342	GLU 340	TRP 348	HOH 785
480865	Licoricidin	ASP 283, SER 345, ASN 396		ALA 238, TYR 244, PRO 245, LEU 317, LEU 287, LEU 241, LEU 314, PHE 316, TRP 348			HOH 785
11090206	Tokinolide B	GLU 235		PHE 246, PRO 245, TYR 244, TYR 313, TRP 348, LEU 241, ALA 238		SER 345	
155289224	Pyrrolopyrazine		PHE 246, TRP 348	LEU 314, TYR 313, PRO 245	GLU 349	SER 345, ASN 396, TRS 507	

**Table 4:** Predicted pharmacokinetic properties and drug-likeness assessment of selected phytochemicals of *X. xylocarpa* based on Lipinski filter and ADMET profiling.

No	PubChem ID	Compounds	ADMETLab	QikProp							
			HEP *	HOA *	SOL *	BBB *	HAS *	LogP *	MW *	HBD *	HBA *
			ADMET					Lipinski			
1	9547215	Bufalin	0.13	3	−4.97	−0.68	0.71	3.52	386.53	2	5
2	10181133	Cerevisterol	0.11	1	−6.65	−0.87	1.25	5.47	430.67	3	4
3	480865	Licoricidin	0.74	1	−6.77	−0.97	1.10	5.42	424.53	3	4
4	158048	Senbusine A	0.07	3	−1.85	0.25	−0.35	0.91	423.54	4	7
5	11090206	Tokinolide B	0.41	3	−4.84	−0.68	0.44	3.92	380.48	0	6
6	15543012	Vitetrifolin C	0.75	3	−5.19	−0.28	0.92	4.86	360.49	1	3
**Reference value**			<0.30 low	1 = Low 2 =Medium 3 = High	>−4 Good −6 to −4 Medium <−6 Poor	>0.3 High −1.0 to 0.3 Medium <−1.0 Poor	<−1.0 Low −1.0 to 0.5 Medium >0.5 High	≤5	≤500	≤5	≤10

* HEP: hepatotoxicity; HOA: human oral absorption; SOL: solubility; BBB: blood-brain barrier; HSA: human serum albumin; LogP: Octanol-water partition coefficient; MW: molecular weight; HBD: hydrogen bond donor; HBA: hydrogen bond acceptor.

**Table 5:** PASS-predicted pharmacological activity profile of Senbusine A, indicating potential neuroactive and receptor-modulating properties.

Pa *	Pi *	Activity
0.97	0.002	Anesthetic
0.92	0.002	Anesthetic local
0.89	0.004	Analgesic
0.86	0.003	Acetylcholine antagonist
0.86	0.003	Cholinergic antagonist
0.84	0.001	Acetylcholine nicotinic antagonist

* Pa: Probability of activity; Pi: probability of inactivity.

## Data Availability

The original contributions presented in the study are included in the article/Supplementary Material, further inquiries can be directed to the corresponding author.

## Supplementary Materials

The following supporting information can be downloaded at: https://www.mdpi.com/article/10.3390/plants15111731/s1, Table S1: Tentatively annotated metabolites detected in Xylia xylocarpa extract by UPLC-QTOF-MS in positive (ESI+) and negative (ESI−) ionization modes.
