# Supplementary material for: Structure-Based Identification of Allosteric Glucocerebrosidase Stabilizers from Xylia xylocarpa (Roxb.) Taub. for Parkinson’s Disease Using LC-MS Profiling and Computational Analysis
Source: Plants (Basel). 2026 Jun 3;15(11):1731. doi: 10.3390/plants15111731 (PMC13258993; doi:10.3390/plants15111731)
Supplement: Supplementary file 1 [file plants-15-01731-s001.zip › plants-4254191-supplementary/Supplementary Table S1.pdf]

Supplementary Table S1: Tentatively annotated metabolites detected in Xylia xylocarpa extract by UPLC–QTOF–MS in positive (ESI^+) and negative (ESI^-) ionization modes

Sl. No.	Compound name	RT (min)	Observed m/z	Adduct	Ion mode	Neutral mass (Da)	Compound class
1	Acetoxy-[10]-gingerol	12.90	413.2648	+Na	ESI^+	390.27	Phenolic ketone
2	Senbusine A	13.73	441.2944	$+NH_4$	ESI^+	423.26	Alkaloid
3	Bufalin	10.43	425.2102	+K	ESI^+	386.25	Steroidal terpenoid
4	Cerevisterol	16.07	430.3447	+H	ESI^+	430.34	Sterol
5	Licoricidin	4.93	447.2129	+Na	ESI^+	424.22	Isoflavonoid
6	Tokinolide B	4.81	403.1899	+Na	ESI^+	380.20	Sesquiterpene lactone
7	Vitetrifolin C	6.18	399.1931	+K	ESI^+	360.23	Diterpenoid
8	Methyl nigakinone	4.59	319.0483	+K	ESI^+	280.09	Naphthoquinone
9	Hexadecyl ferulate	13.73	419.3140	+H	ESI^+	418.31	Phenolic ester
10	12-Oxoarundoin	14.42	454.3810	+Na/+K	ESI^+	454.38	Triterpenoid derivative
11	ent-12α,16-Epoxy-pimarene derivative	11.37	337.2384	+H	ESI^+	336.23	Diterpenoid
12	Sarmentoloside	4.43	569.2943	+H	ESI^+	568.29	Glycoside
13	Picrasinoside D	12.91	621.2869	+Na	ESI^+	598.30	Terpenoid glycoside
14	Celosin E	11.87	679.3671	+H	ESI^+	678.36	Triterpenoid glycoside
15	Platycoside K	4.07	453.2012	+Na/K	ESI^+	844.45	Saponin glycoside
16	Epimedin C	4.57	463.1516	+HCOO	ESI^-	822.29	Flavonoid glycoside
17	14,17-Octadecadienoic acid	13.97	325.2395	+HCOO	ESI^-	280.24	Fatty acid
18	2-Cyclopentene-1-undecanoic acid	10.66	311.2222	$+CH_3COO$	ESI^-	252.21	Fatty acid derivative
19	1-Heptatriacontanol	8.50	313.2920	+HCOO	ESI^-	536.59	Long-chain alcohol
